# Cross-State Travel for Cancer Care and Implications for Telehealth Reciprocity

**DOI:** 10.1001/jamanetworkopen.2024.61021

**Published:** 2025-02-21

**Authors:** Erika L. Moen, Qianfei Wang, Lingbo Liu, Fahui Wang, Anna N. A. Tosteson, Rebecca E. Smith, Lauren Cowan, Tracy Onega

**Affiliations:** 1 Department of Biomedical Data Science, Geisel School of Medicine at Dartmouth, Lebanon, New Hampshire; 2 The Dartmouth Institute for Health Policy and Clinical Practice, Geisel School of Medicine at Dartmouth, Lebanon, New Hampshire; 3 Dartmouth Cancer Center, Geisel School of Medicine at Dartmouth, Lebanon, New Hampshire; 4 Center for Geographic Analysis, Harvard University, Cambridge, Massachusetts; 5 Department of Geography and Anthropology, Louisiana State University, Baton Rouge; 6 Huntsman Cancer Institute, University of Utah, Salt Lake City; 7 Department of Population Health Sciences, University of Utah, Salt Lake City

## Abstract

**Importance:**

Patients often travel for cancer care, yet the extent to which patients cross state lines for cancer care is not well understood. This knowledge can have implications for policies that regulate telehealth access to out-of-state clinicians.

**Objective:**

To quantify the extent of cross-state delivery of cancer services to patients with cancer.

**Design, Setting, and Participants:**

This cross-sectional study analyzed fee-for-service Medicare claims data for beneficiaries (aged ≥66 years) with a diagnosis of breast, colon, lung, or pancreatic cancer between January 1, 2017, and December 31, 2020. Analyses were performed between January 1 and July 30, 2024.

**Exposure:**

Patient rurality.

**Main Outcomes and Measures:**

The primary outcome of interest was receipt of cancer care across state lines. Frequencies of cancer services (surgery, radiation, and chemotherapy) were summarized by cancer type in relation to in-state vs out-of-state receipt of care based on state of residence for Medicare beneficiaries. Cross-state delivery of cancer services was also quantified by adjacent vs nonadjacent states and overall between-state flows for service utilization.

**Results:**

The study included 1 040 874 Medicare beneficiaries with cancer. The mean (SD) age of the study population was 76.5 (7.4) years. Most patients were female (68.2%) and urban residing (78.5%); one-quarter (25.9%) were aged between 70 and 74 years. In terms of race and ethnicity, 7.0% of patients identified as Black, 3.4% as Hispanic, and 85.5% as White. Overall, approximately 6.9% of cancer care was delivered across state lines, with the highest proportion (8.3%) occurring for surgical care, followed by radiation (6.7%) and chemotherapy (5.6%) services. Out of all cross-state care, 68.4% occurred in adjacent states. Frequency of cross-state cancer care increased with patient rurality. Compared with urban-residing patients, isolated rural-residing patients were 2.5 times more likely to cross state lines for surgical procedures (18.5% vs 7.5%), 3 times more likely to cross state lines for radiation therapy services (16.9% vs 5.7%), and almost 4 times more likely to cross state lines for chemotherapy services (16.3% vs 4.2%).

**Conclusions and Relevance:**

In this cross-sectional study of Medicare claims data, a notable proportion of cancer services occurred across state lines, particularly for rural-residing patients. These results highlight the need for cross-state telehealth policies that recognize the prevalence of care delivery from geographically distant specialized oncology services.

## Key Points

**Question:**

What are the implications of out-of-state cancer care delivery for cross-state telehealth policies?

**Findings:**

In this cross-sectional study of 1 040 874 Medicare beneficiaries with cancer, approximately 7% of cancer care was delivered across state lines. Compared with urban-residing patients, isolated rural-residing patients were approximately 2 times more likely to cross state lines for surgical procedures (19% vs 8%), 3 times more likely to cross state lines for radiation therapy services (17% vs 6%), and almost 4 times more likely to cross state lines for chemotherapy services (16% vs 4%).

**Meaning:**

These findings suggest that the prevalence of cross-state oncology care underscores the importance of cross-state telehealth policies, particularly for rural-residing patients.

## Teaser

This cross-sectional study explores the extent of cancer care delivery across state lines for Medicare beneficiaries and describes implications for cross-state telehealth policies.

## Introduction

Geographic barriers to cancer care are well studied and often captured with measures of travel distance or travel time to the nearest or actual facility where cancer care is provided.^1,2,3,4^ Yet access to cancer care is rapidly evolving to encompass care delivered via telehealth.^5^ Telehealth in cancer care can facilitate treatment follow-up consultations, management of treatment-related toxicities, and consultations related to screening and midcycle visits for clinical trials.^6^ Access to telehealth can be especially beneficial for patients with travel limitations or those who have greater travel burden to care.^7^ Cross-state policies for telehealth govern the extent to which residents of a state have access to out-of-state clinicians via telehealth.^8^ Barriers to accessing cancer care can be compounded when patients face geographic barriers to in-person care and policy limitations to telehealth use with oncologists who are out of state, motivating alignment of practice and policy.^9^

Although cross-state policy restrictions were rolled back during the COVID-19 pandemic, all waivers expired at the end of 2023, and several states either ban or severely restrict telehealth appointments with clinicians licensed out of state.^10^ Prior work has shown lower use of telehealth among patients with cancer who live in states with cross-state policy restrictions compared with those who live in states with no restrictions.^11^ A better understanding of the extent to which cross-state cancer care is being delivered across patient subgroups and stratified by cancer services can help guide cross-state policy to ensure adequate and equitable access to care.

The objective of this study was to quantify the extent of cross-state delivery of cancer services to patients with cancer in the US in a largely prepandemic context. We hypothesized that among a sample of original Medicare beneficiaries with breast, colon, lung, or pancreatic cancer newly diagnosed between 2017 and 2020, the frequency of cross-state travel would increase with specialization of procedure and rurality of residence. We examined the frequency of cross-state cancer care for cancer-directed surgery, chemotherapy, and radiation therapy by cancer type, patient race and ethnicity, and rurality.

## Methods

The University of Utah Institutional Review Board deemed this cross-sectional study exempt from further review because claims data were used, and a waiver of informed consent was granted. The study followed the Strengthening the Reporting of Observational Studies in Epidemiology (STROBE) reporting guideline.

### Data Source and Study Cohort

We obtained Centers for Medicare & Medicaid Services (CMS) Medicare enrollment and claims data from January 1, 2017, to December 31, 2020, for this study. We implemented a published method, modified for use with *International Statistical Classification of Diseases, Tenth Revision, Clinical Modification* (*ICD-10-CM*) diagnosis codes, to identify beneficiaries with an incident diagnosis of breast, colon, lung, or pancreatic cancer from the 100% sample of fee-for-service Medicare claims (eTable in Supplement 1).^12^ Patients were excluded if they did not have continuous enrollment in Medicare Parts A and B to the end of 2020 or up to death, whichever came first. Patients were further excluded if they were younger than 66 years at the time of cohort eligibility or had a missing or non-US zip code. We also excluded patients who had end-stage kidney disease or were enrolled in a health maintenance organization (HMO). We excluded individuals enrolled in HMOs because the CMS does not provide their complete claims data. We excluded patients with a cancer diagnosis code in the 12 months preceding their index date to enrich for incident cancer cases.

### Study Variables

Patient age, race and ethnicity, and zip code and state of residence were identified in the Master Beneficiary Summary File, which includes the Research Triangle Institute’s algorithm for derived race. Race and ethnicity were examined to assess the potential implications for equitable access to telehealth services based on differential frequencies of cross-state cancer care utilization. These data are reported as Asian, Black, Hispanic, White, or other race or ethnicity (categorized as American Indian or Alaska Native or unknown or other race or ethnicity not specified due to cell suppression policies). Residential zip codes were linked to Rural-Urban Commuting Area (RUCA) codes and used to assign the 4-tiered rural categorization to patients in our cohort to preserve sufficient variability while maintaining analytic simplicity.^13^ We used the Washington University Classification system and assigned zip codes as urban (1.0, 1.1, 2.0, 2.1, 3.0, 4.1, 5.1, 7.1, 8.1, and 10.1), large rural city (4.0, 4.2, 5.0, 5.2, 6.0, and 6.1), small rural city (7.0, 7.2, 7.3, 7.4, 8.0, 8.2, 8.3, 8.4, 9.0, 9.1, and 9.2), and isolated small town (10.0, 10.2, 10.3, 10.4, 10.5, and 10.6).

### Statistical Analysis

#### Classification of Out-of-State Cancer Services

We used previously published *ICD-10-CM* and *Current Procedural Terminology* codes to identify claims in the CMS Carrier, Outpatient, and MedPAR (Medicare Provider Analysis and Review) files for cancer-directed surgeries, chemotherapy, and radiation therapy (eTable in Supplement 1). For each cancer service claim, a binary flag was created to indicate whether the cancer service was delivered in a different state than the state of residence for the patient. Of the cross-state encounters, we further specified whether the states were adjacent states, which we defined as sharing a common border. Frequencies of cross-state travel for surgery, chemotherapy, and radiation therapy encounters were calculated overall and by cancer type, patient race and ethnicity, and patient rurality.

#### Geospatial Visualization of Cross-State Cancer Services

We used the centroids of each state to represent the locations of regional flows for cross-state cancer services. These flows are directional and are depicted using right-bend Bézier curves, where the movement from the origin to the destination always starts from the right side of the origin. This method enhances the intuitiveness of the flow direction between 2 points. Arrows indicate the direction of travel from state of residence to state of cancer service provided. The thickness of the flow lines is classified into 5 levels using the natural breaks method, providing a clearer representation of distribution patterns.

Alaska and Hawaii were rescaled and repositioned in the visualization to provide a more compact and clear depiction of their flow characteristics. All data processing was conducted using the KNIME Analytics Platform, version 5.3 (KNIME) to ensure reproducibility, and the final visualization was completed in ArcGIS Pro, version 3.1 (Esri).^14^

Statistical analysis was performed using SAS, version 9.4M8 (SAS Institute Inc). Analyses were performed between January 1 and July 30, 2024.

## Results

Our study included 1 040 874 Medicare beneficiaries with an incident diagnosis of breast cancer (377 422 [36.3%]), colon cancer (217 711 [20.9%]), lung cancer (354 884 [34.1%]), or pancreatic cancer (90 857 [8.7%]) (Table 1). The mean (SD) age of the study population was 76.5 (7.4) years. A total of 710 035 patients (68.2%) were female and 330 839 (31.8%) were male. One-quarter of patients (269 319 [25.9%]) were aged between 70 and 74 years, and most (817 348 [78.5%]) were urban residing. Patients identified as Asian (20 080 [1.9%]), Black (72 842 [7.0%]), Hispanic (35 466 [3.4%]), White (890 214 [85.5%]), or other race or ethnicity (22 272 [2.1%]). The total number of surgical procedures, radiation treatments, and chemotherapy treatments delivered to patients in our study cohort stratified by cancer type is reported in Table 2. All subsequent analyses are encounter-level analyses.

**Table 1.** Characteristics of Medicare Beneficiaries With Incident Cancer Diagnoses, Stratified by Cancer Type, 2017-2020^a^

Characteristic	Cancer type			
	Breast (n = 377 422)	Colon (n = 217 711)	Lung (n = 354 884)	Pancreas (n = 90 857)
Sex				
Female	372 582 (98.7)	113 050 (51.9)	178 374 (50.1)	46 029 (50.7)
Male	4840 (1.3)	104 661 (48.1)	176 510 (49.9)	44 828 (49.3)
Age at diagnosis, y				
66-69	86 597 (22.9)	39 957 (18.4)	60 596 (17.1)	15 938 (17.5)
70-74	105 077 (27.8)	46 634 (21.4)	94 989 (26.8)	22 619 (24.9)
75-79	79 693 (21.1)	44 736 (20.6)	85 847 (24.2)	20 296 (22.3)
80-84	53 054 (14.1)	38 838 (17.8)	61 304 (17.3)	15 880 (17.5)
≥85	53 001 (14.0)	47 546 (21.8)	52 148 (14.7)	16 124 (17.8)
Race and ethnicity				
Asian	6786 (1.8)	4851 (2.2)	6250 (1.8)	2193 (2.4)
Black	25 841 (6.9)	16 644 (7.6)	23 198 (6.5)	7159 (7.9)
Hispanic	12 646 (3.4)	9335 (4.3)	9672 (2.7)	3813 (4.2)
White	324 100 (85.9)	181 911 (83.6)	308 864 (87.0)	75 339 (82.9)
Other^b^	8049 (2.1)	4970 (2.3)	6900 (1.9)	2353 (2.6)
Rurality				
Urban	303 207 (80.3)	167 254 (76.8)	274 052 (77.2)	72 835 (80.2)
Large rural city	38 752 (10.3)	25 283 (11.6)	41 510 (11.7)	9178 (10.1)
Small rural town	20 548 (5.4)	14 626 (6.7)	22 954 (6.5)	5107 (5.6)
Isolated small rural town	14 915 (4.0)	10 548 (4.8)	16 368 (4.6)	3737 (4.1)

^a^ Data are presented as No. (%) of beneficiaries.

^b^ Includes American Indian or Alaska Native, other race or ethnicity not specified, and unknown race or ethnicity.

**Table 2.** Cancer Services Provided to Medicare Beneficiaries, Stratified by Cancer Type^a^

Cancer service type	Cancer type			
	Breast	Colon	Lung	Pancreas
Surgery	466 811 (9.7)	212 504 (23.2)	137 449 (9.0)	28 355 (5.2)
Radiation	1 924 420 (72.6)	232 831 (25.4)	757 023 (48.8)	143 936 (26.6)
Chemotherapy	257 814 (17.6)	471 332 (51.4)	634 371 (42.2)	369 818 (68.2)

^a^ Data are presented as No. (%) of beneficiaries.

### Cross-State Travel by Cancer Site and Service Type

**Figure 1.**
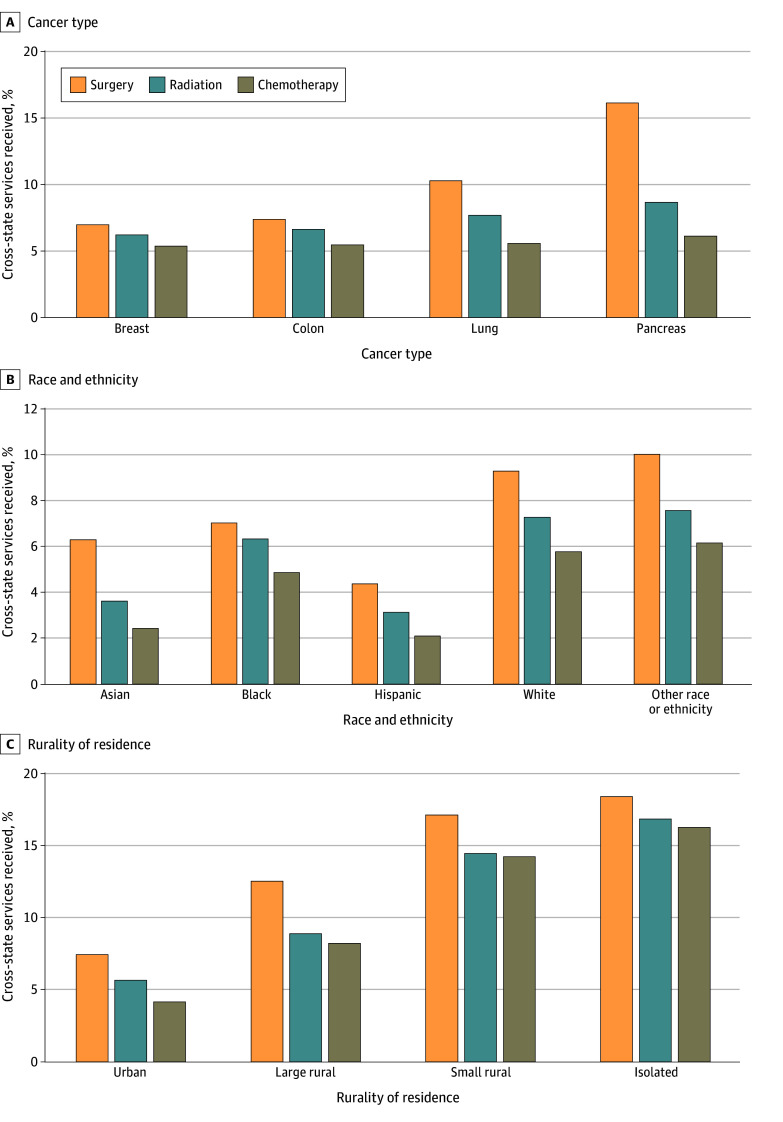
Services Received Across State Lines Out of Total Services Percentages were calculated separately for surgical procedures, radiation therapy, and chemotherapy stratified by cancer type, patient race and ethnicity, and rurality of residence.

Of cancer services delivered to patients in our cohort, 8.3% of surgical procedures, 6.7% of radiation therapy, and 5.6% of chemotherapy services were received across state lines. Overall, approximately 6.9% of cancer care was delivered across state lines. The frequencies of cross-state travel for surgery, radiation therapy, and chemotherapy varied across cancer types (Figure 1A). Cross-state cancer services were least frequent among patients with breast cancer, with 7.0% of surgeries, 6.2% of radiation therapy services, and 5.4% of chemotherapy services received out of state. Patients with pancreatic cancer had the highest frequencies of cross-state cancer services, with 16.2% of surgeries, 8.7% of radiation therapy services, and 6.1% of chemotherapy services received out of state. Cross-state travel frequencies for colon and lung cancer fell between them and showed similar trends, with surgeries most frequently and chemotherapy services least frequently received across state lines. Of cancer services received across state lines, 73.7% of surgical procedures, 67.7% of radiation therapy services, and 64.3% of chemotherapy services occurred in adjacent states (eFigure 1 in Supplement 1). Out of all cross-state care, 68.4% occurred in adjacent states.

**Figure 2.**
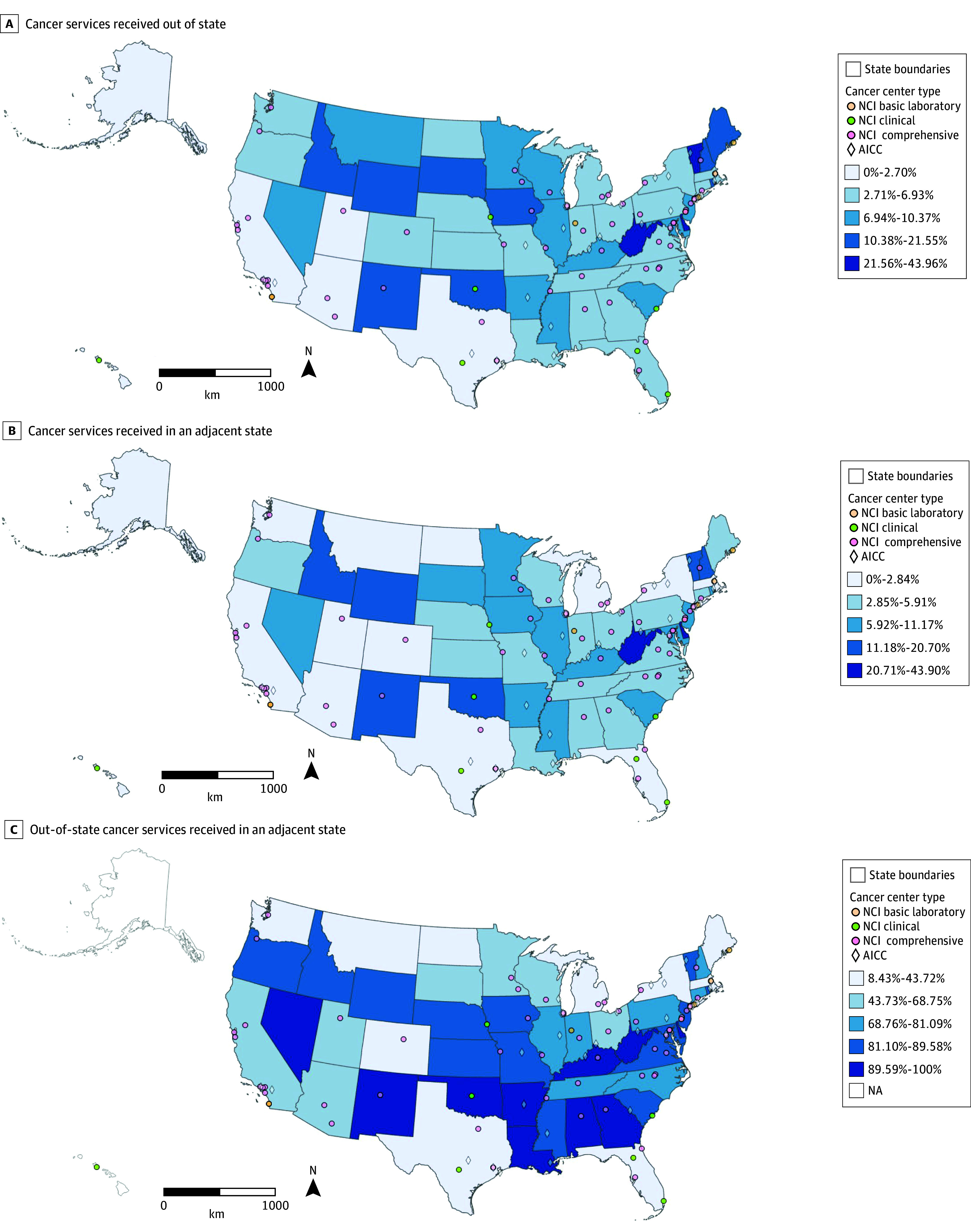
State-Level Proportions of Cross-State Travel for Cancer Care AACI indicates Association of American Cancer Institutes; NCI, National Cancer Institute.

We visualized state-level variation in the percentage of total cancer services received out of state (Figure 2A) and in adjacent states (Figure 2B). Delaware, Vermont, West Virginia, and the District of Columbia had the highest rates of out-of-state travel (≥21.56%). States with the highest percentage (≥89.59%) of outgoing cross-state travel occurring in adjacent states included Nevada, New Mexico, Oklahoma, Arkansas, Alabama, Louisiana, Georgia, West Virginia, Kentucky, and Delaware (Figure 2C). Those with the lowest percentage (≤43.72%) of outgoing cross-state travel occurring in adjacent states included Texas, Florida, and Montana, indicating most cross-state travel for cancer services was to nonadjacent states. Alaska and Hawaii have no adjacent states, so all observed cross-state cancer services were considered nonadjacent (Figure 2C).

Flow of cancer services between all pairs of states was visualized for overall cancer services, surgical procedures, radiation therapy, and chemotherapy (eFigure 2 in Supplement 1). Across all 4 visualizations, we observed instances of long-distance patient movement. There was a consistent pattern of high-volume cancer service flows along the East Coast, particularly between the Northeastern and Southeastern states. The tendency to travel to adjacent or nearby states was more prominent in the Midwestern and Southern states.

### Cross-State Travel by Patient Race and Ethnicity, Patient Rurality, and Service Type

Across all cancers, there was variation in the percentage of cancer services received out of state by patient race and ethnicity and patient rurality of residence (Figure 1B and C). Overall, White patients had the highest percentage of cancer services received across state lines, with 9.3% of surgeries, 7.3% of radiation therapy services, and 5.8% of chemotherapy services received out of state (Figure 1B). Cross-state travel was least frequent among Hispanic patients, with 4.4% of surgeries, 3.1% of radiation therapy services, and 2.1% of chemotherapy services received across state lines. We found that cancer services to rural-residing patients were 2 to almost 4 times more likely to be delivered across state lines compared with those delivered to urban-residing patients (Figure 1C). Among urban-residing patients, 7.5% of surgeries, 5.7% of radiation therapy services, and 4.2% of chemotherapy services were received out of state. Among the most rural-residing patients (isolated tier of rurality), 18.5% of surgeries, 16.9% of radiation therapy services, and 16.3% of chemotherapy services were received out of state. Frequencies of cross-state travel for cancer services among large rural-residing and small rural-residing patients followed similar trends, increasing with rurality of residence.

The greater frequency of cross-state travel for cancer services by patient rurality of residence were largely consistent when analyzed by cancer type (Table 3). The frequency of cross-state travel for rural-residing patients was particularly notable for lung and pancreatic cancer surgical procedures, with 20.0% of small-town rural-residing and 21.2% of isolated rural-residing patients with lung cancer receiving surgical procedures out of state, and 27.4% of small-town rural-residing and 26.0% of isolated rural-residing patients with pancreatic cancer receiving surgical procedures out of state.

**Table 3.** Frequency of Cross-State Cancer Service Delivery Out of Total Services by Patient Race and Ethnicity and Rurality^a^

Cohort	Cross-state service		
	Surgery	Radiation therapy	Chemotherapy
**Breast cancer**			
Total services	257 814	1 924 420	466 811
Cross-state services	18 027	119 828	25 131
Race and ethnicity			
Asian	241 (5.1)	1242 (4.0)	291 (3.0)
Black	1011 (6.4)	7169 (5.8)	1943 (5.2)
Hispanic	320 (4.1)	2050 (3.3)	352 (1.9)
White	15 982 (7.2)	106 073 (6.4)	21 909 (5.6)
Other^b^	473 (8.0)	3294 (7.5)	636 (6.3)
Rurality			
Urban	11 779 (5.7)	76 686 (5.0)	16 299 (4.2)
Large rural city	2612 (9.6)	17 280 (8.6)	3367 (7.8)
Small rural town	2017 (14.2)	13 862 (14.0)	2833 (14.0)
Isolated small rural town	1619 (15.1)	12 000 (16.2)	2632 (15.9)
**Colon cancer**			
Total services	212 504	232 831	471 332
Cross-state services	15 725	15 487	25 711
Race and ethnicity			
Asian	222 (5.4)	85 (1.5)	280 (2.6)
Black	881 (6.5)	1071 (7.3)	1639 (4.8)
Hispanic	296 (3.7)	216 (1.7)	540 (2.4)
White	13 961 (7.7)	13 801 (7.1)	22 467 (5.8)
Other^b^	365 (7.6)	314 (6.5)	785 (5.7)
Rurality			
Urban	9647 (6.0)	9390 (5.3)	15 274 (4.0)
Large rural city	2438 (9.4)	2058 (7.4)	3779 (7.9)
Small rural town	1949 (13.3)	2132 (13.0)	3531 (13.1)
Isolated small rural town	1691 (15.4)	1907 (17.5)	3127 (16.0)
**Lung cancer**			
Total services	180 262	976 772	844 363
Cross-state services	18 250	74 665	47 162
Race and ethnicity			
Asian	268 (7.3)	630 (4.5)	266 (2.1)
Black	665 (7.7)	3514 (6.0)	2316 (4.6)
Hispanic	188 (4.2)	803 (3.6)	635 (2.8)
White	16 648 (10.6)	68 151 (7.9)	42 892 (5.8)
Other^b^	481 (11.2)	1567 (8.2)	1053 (6.4)
Rurality			
Urban	12 258 (8.4)	46 803 (6.2)	27 910 (4.1)
Large rural city	2724 (15.0)	11 098 (9.4)	7195 (8.4)
Small rural town	1819 (20.0)	9287 (14.7)	6622 (14.9)
Isolated small rural town	1449 (21.2)	7477 (16.7)	5435 (16.6)
**Pancreatic cancer**			
Total services	8355	143 936	369 818
Cross-state services	4580	12 462	22 664
Race and ethnicity			
Asian	88 (12.9)	185 (5.6)	190 (2.2)
Black	154 (9.7)	681 (7.4)	1297 (5.8)
Hispanic	73 (6.3)	292 (4.2)	259 (1.6)
White	4105 (17.1)	10 958 (9.1)	20 337 (6.5)
Other^b^	160 (18.4)	346 (9.0)	581 (5.9)
Rurality			
Urban	3334 (14.3)	8756 (7.4)	14 489 (4.7)
Large rural city	577 (23.0)	1528 (11.9)	2944 (9.6)
Small rural town	389 (27.4)	1375 (18.6)	2606 (16.8)
Isolated small rural town	280 (26.0)	803 (17.3)	2625 (19.6)

^a^ Data are presented as No. (%) of patients.

^b^ Includes American Indian or Alaska Native, other race or ethnicity not specified, and unknown race or ethnicity.

## Discussion

In this study of nationwide Medicare claims data from 2017 to 2020, we quantified the amount of cancer care delivered across state lines. Overall, we observed that cross-state travel for cancer services varied by cancer type, rural residency, and patient race and ethnicity. Across all cancer types, cross-state travel was most frequent for surgical procedures, with the highest frequencies observed for lung and pancreatic cancer surgical procedures. Previous studies suggested that regionalization of specialized services, such as complex cancer surgeries, may improve outcomes by funneling patients to high-volume health care professionals.^15,16^ National Cancer Institute Comprehensive Cancer Centers often serve as regional hubs for cancer care, and we expect that the locations of these centers are a contributing factor to care delivery across state lines. Lung and pancreatic cancer surgical procedures are more likely to be regionalized than breast or colon cancer procedures due to complexity, risk of complications, and setting of care.^17^ However, concerns have been raised that regionalization of care places substantial travel burden on patients, particularly those who reside in rural areas, which our results corroborate.^18,19^ Across the cancer treatment services examined in this study, chemotherapy was the least likely to be delivered across state lines for all cancer types, which is anticipated given the high cumulative travel burden for receiving multiple cycles of chemotherapy over the course of treatment. Although delivery of cancer treatment requires in-person care, telehealth can facilitate preoperative and postoperative consultations and visits to discuss symptom management during adjuvant care. As such, cross-state telehealth policies can affect a patient’s ability to access their specialists using telehealth for consultations that support care continuity and quality of life.^20^

Our comprehensive geospatial analysis of cross-state cancer services in the US provides a holistic view of how geography is associated with cancer care accessibility, revealing notable patterns of patient movement across state lines and consistent with the draw of regional health care hubs. We also observed the so-called snowbird effect, which is a term used to describe the seasonal migration of retirees from the Northeastern and Midwestern parts of the US to Florida. In this study, the snowbird effect may explain some of the cross-state travel observed.

We were particularly struck by the 2- to almost 4-fold increase in cross-state travel for cancer care among rural-residing patients compared with urban-residing patients, with frequencies of 18.5% vs 7.5% for surgical procedures, 16.9% vs 5.7% for radiation therapy services, and 16.3% vs 4.2% for chemotherapy services. Furthermore, urban-residing patients in this study had a greater relative decrease in cross-state travel for chemotherapy and radiation therapy compared with surgical procedures, which suggests that preference or ability to travel greater distances for specialized services, such as surgical procedures, is more prevalent among urban-residing patients. The smaller relative changes in cross-state travel for cancer care across service types observed among rural-residing patients suggest that the travel was more likely out of necessity (ie, lack of local options). Given that cross-state policies for telehealth can enhance care delivery from geographically distant clinicians, it is important to consider how telehealth policies that restrict access to physicians licensed in another state may disparately affect patients who are already facing additional geographic barriers to care.

We found notable variation across states in the percentage of cross-state travel that occurred in an adjacent state, which we defined as having a common border. Licensing across state lines is regulated by state policies, and some states allow clinicians from another state to provide telehealth services if they share a common border. Our findings increase understanding of the extent to which cross-state travel for cancer care is being delivered in adjacent states, which can be used to determine the states that would most benefit from licensure reciprocity with adjacent states. Multistate licensing compacts, which are created when states agree on a uniform standard of care and enact state laws, may be more effective at ensuring access to telehealth among states where a lower percentage of cross-state travel is occurring in adjacent states or for states that share borders with few other states.

Our analysis of cross-state travel for cancer care also has broader implications for care fragmentation. Fragmentation of care has previously been defined as receiving care at more than 1 institution and is prevalent in cancer treatment.^21,22^ Relevant to our study, care fragmentation may occur among patients who cross state lines for surgical treatment and then receive chemotherapy services closer to home. The role of telehealth in facilitating virtual communication between patients and out-of-state health care professionals may mitigate some of the concerns associated with care fragmentation, such as communication gaps and decreased patient satisfaction.^23,24^ Future work examining the interplay between care fragmentation, telehealth use, and patient care experiences and outcomes are important next steps, particularly for patients who receive part or all of their treatment in another state.

### Limitations

This study has several limitations. First, because our cohort was limited to fee-for-service Medicare beneficiaries, our findings may not be generalizable to other populations, such as younger patients who may be more able to travel or to patients with other cancer types. Our analyses did not include beneficiaries enrolled in Medicare Advantage. Up to 54% of eligible Medicare beneficiaries are enrolled in Medicare Advantage, with enrollment varying substantially by state. Prior work has shown that patients with cancer enrolled in Medicare Advantage have lower access to high-volume hospitals, which may affect the likelihood of patients traveling out of state for cancer services.^25,26^ Future work is needed to better understand the role of insurance plan type in cross-state travel for cancer care. Second, we determined cross-state care using beneficiary zip code of residence, which may not reflect the location of where individuals were living during treatment for those who have second homes or stayed with family. We also used the RUCA 4-tier classification to define rurality based on zip code of residence, yet many standardized definitions of urban and rural exist.^27^ Third, because study data from 2017 to 2019 predated the COVID-19 pandemic, changes such as workforce shifts, travel restrictions, reduced cancer diagnosis rates, and expanded telehealth may affect how our findings apply to current cross-state travel for cancer care. Finally, patients receiving out-of-state care are likely a mix of those doing so out of preference and those doing so out of necessity due to limited local options. On one hand, it is possible that having an out-of-state surgical procedure may be reflecting patient preference or ability to travel to a regional or national center. On the other hand, out-of-state adjuvant care that requires many trips for cycles of treatment may be more likely reflecting a lack of limited local options. Although we were not able to capture patient preference in these analyses, this is an important future direction for research.

## Conclusions

In this cross-sectional study, we observed that a notable proportion of cancer service delivery occurred across state lines, particularly for rural-residing patients. Our findings suggest that as telehealth use is integrated into care pathways for patients with cancer, policy in this realm should be aligned with practice. These findings also highlight possible inequities in the effects of cross-state telehealth policies on access to telehealth services for patients with cancer. It is critical that cross-state telehealth policies recognize the need to access specialized physicians, who may be more geographically distant, by telehealth.

## Notes

Supplement 1. **eTable.** Diagnosis and Procedure Codes Used to Define Cohort and Cancer Services **eFigure 1.** Sankey Diagram Visualizing Flow of Radiation, Chemotherapy, and Surgical Procedures to Adjacent and Nonadjacent States **eFigure 2**. Between-State Flows of Cancer Services for All Cancer Services (A), Surgical Procedures (B), Radiation Therapy (C), and Chemotherapy (D)

Supplement 2.
Data Sharing Statement
