# Supplementary material for: Cross-State Travel for Cancer Care and Implications for Telehealth Reciprocity
Source: JAMA Netw Open. 2025 Feb 21;8(2):e2461021. doi: 10.1001/jamanetworkopen.2024.61021 (PMC11846006; doi:10.1001/jamanetworkopen.2024.61021)
Supplement: Supplement 1. — eTable. Diagnosis and Procedure Codes Used to Define Cohort and Cancer Services eFigure 1. Sankey Diagram Visualizing Flow of Radiation, Chemotherapy, and Surgical Procedures to Adjacent and Nonadjacent States eFigure 2. Between-State Flows of Cancer Services for All Cancer Services (A), Surgical Procedures (B), Radiation Therapy (C), and Chemotherapy (D) [file jamanetwopen-e2461021-s001.pdf]

Supplementary Online Content

Moen EL, Wang Q, Liu L, et al. Cross-state travel for cancer care and implications for telehealth reciprocity. *JAMA Netw Open*. 2025;8(2):e2461021. doi:10.1001/jamanetworkopen.2024.61021

eTable. Diagnosis and Procedure Codes Used to Define Cohort and Cancer Services

eFigure 1. Sankey Diagram Visualizing Flow of Radiation, Chemotherapy, and Surgical Procedures to Adjacent and Nonadjacent States

eFigure 2. Between-State Flows of Cancer Services for All Cancer Services (A), Surgical Procedures (B), Radiation Therapy (C), and Chemotherapy (D)

This supplementary material has been provided by the authors to give readers additional information about their work.

© 2025 Moen EL et al. *JAMA Network Open*.

eTable. Diagnosis and Procedure Codes Used to Define Cohort and Cancer Services

**Breast cancer codes**	
Diagnosis	ICD-10: C50
Biopsy	CPT: 10021, 10022, 19000, 19001, 19081, 19082, 19083, 19084, 19085, 19086, 19100, 19101, 19120, 19125, 19126, 19281, 19282, 19283, 19284, 19285, 19286, 19287, 19288<br>ICD-10: 0HBT0ZX, 0HBT3ZX, 0HBT4ZX, 0HBT7ZX, 0HBT8ZX, 0HBU0ZX, 0HBU3ZX, 0HBU4ZX, 0HBU7ZX, 0HBU8ZX, 0HBV0ZX, 0HBV3ZX, 0HBV4ZX, 0HBV7ZX, 0HBV8ZX
Surgery	CPT: 19112, 19120, 19125, 19126, 19160, 19162, 19180, 19182, 19200, 19220, 19240, 19301, 19302, 19303, 19304, 19305, 19306, 19307<br>ICD-10: 0HBT0ZZ, 0HBT3ZZ, 0HBT7ZZ, 0HBT8ZZ, 0HBU0ZZ, 0HBU3ZZ, 0HBU7ZZ, 0HBU8ZZ, 0HBV0ZZ, 0HBV3ZZ, 0HBV7ZZ, 0HBV8ZZ
Chemotherapy	CPT: 36640, 51720, 96401, 96405, 96406, 96408, 96410, 96412, 96414, 96420, 96422, 96423, 96425, 96440, 96445, 96450, 96501, 96504, 96505, 96508, 96510, 96511, 96512, 96520, 96524, 96530, 96538, 96540, 96542, 96545, 96549, 96450, 96555<br>HCPCS: J0202, J1675, J1930, J1950, J2353, J2354, J2860, J3315, J3316, J7504, J7511, J8527, J8520, J8530, J8560, J8565, J8565, J8600, J8705, J8999, J9000-J9999
Radiation therapy	CPT: 77261, 77262, 77263, 77280, 77281, 77282, 77283, 77284 77285, 77286, 77287, 77289 77290, 77295, 77300, 77301, 77331, 77338, 77306, 77307, 77316, 77317, 77318, 77321, 77332, 77333, 77334, 77336, 77370, 77761, 77762, 77763, 77767, 77768, 77770, 77771, 77772, 77778, 0394T, 0395T, 77789, 77750, 77790, 77401, 77402, 77403, 77404, 77405, 77406, 77407, 77408, 77409, 77410, 77411, 77412, 77413, 77414, 77415, 77416, 77385, 77386, 77424, 77425, 77422, 77423, 77520, 77521, 77522, 77523, 77524, 77525, 77371, 77372, 77373, 77387, 77014, 77427, 77431, 77432, 77435, 77469, 77470<br>ICD-10: DM00.XX, DM01.XX
**Lung cancer codes**	
Diagnosis	ICD-10: C3400, C3401, C3402, C3410, C3411, C3412, C342, C3430, C3431, C3432, C3480, C3481, C3482, C3490, C3491, C3492, C399
Biopsy	CPT: 32400, 32405, 32604, 31625, 31628, 31629, 31640, 31623, 31624, 31632, 31633, 32096, 32097, 32098, 88305, 88309
Surgery	CPT: 32440, 32442, 32445, 32480, 32482, 32484, 32486, 32488, 32520, 32522, 32525, 32650, 32651, 32652, 32656, 32657, 32659, 32663, 32503, 31641, 32504, 32505, 32506, 32507, 32666, 32667, 32668, 32669, 32670, 32671, 32674
Chemotherapy	CPT: 36640, 51720, 96401, 96402, 96405, 96406, 96409, 96413, 96415, 96416, 96420, 96422, 96423, 96425, 96440, 96446, 96450, 96521, 96522, 96542, 96549<br>ICD-10: Z51.11, Z51.12, 3E03305, 3E04305, XW03351, XW033B3, XW033C3, XW04351, XW043B3, XW043C3<br>HCPCS : Q0083-0085, J9000-J9999<br>Revenue Center 0331, 0332, 0335
Radiation therapy	CPT: 77261, 77262, 77263, 77280, 77285, 77290, 77295, 77299, 77300, 77306, 77307, 77321, 77316, 77317, 77318, 77331, 77332, 77333, 77334,

© 2025 Moen EL et al. *JAMA Network Open*.

	77336, 77370, 77399, 77401, 77402, 77407, 77412, 77417, 77425, 77431, 77432, 77470, 77499, 55875, 55860, 55862, 55865, 77750, 77761, 77762, 77763, 77778, 77770, 77771, 77772, 77789, 77790, 77799, 79200, 79300, 79440, 79999
**Colon cancer codes**	
Diagnosis	ICD-10: C18.0, C18.2, C18.3, C18.4, C18.5, C18.6, C18.7, C18.8, C18.9, C19
Biopsy	CPT: 44100, 45305, 45308, 45309, 45315, 45317, 45320, 45331, 45333, 45338, 45339, 45341, 45342, 45355, 45380, 45383, 45384, 45385, 88305, 88309
Surgery	CPT: 44140, 44141, 44143, 44144, 44145, 44146, 44147, 44150, 44151, 44152, 44153, 44155, 44156, 44160, 44204, 44140, 44205, 44160, 44206, 44143, 44207, 44145, 44208, 44146, 44210, 44150, 44211, 44158, 44212, 44155<br>ICD-10: 0DTE4ZZ, 0DTF4ZZ, 0DTG4ZZ, 0DTH4ZZ, 0DTK4ZZ, 0DTL4ZZ, 0DTM4ZZ, 0DTN4ZZ, 0DTE8ZZ, 0DTF8ZZ, 0DTG8ZZ, 0DTH8ZZ, 0DTK8ZZ, 0DTL8ZZ, 0DTM8ZZ, 0DTN8ZZ, 0DTEFZZ, 0DTFFZZ, 0DTGFZZ, 0DTHFZZ, 0DTKFZZ, 0DTLFZZ, 0DTMFZZ, 0DTNFZZ, 0DTE0ZZ, 0DTF0ZZ, 0DTG0ZZ, 0DTH0ZZ, 0DTK0ZZ, 0DTL0ZZ, 0DTM0ZZ, 0DTN0ZZ, 0DTE7ZZ, 0DTF7ZZ, 0DTG7ZZ, 0DTH7ZZ, 0DTK7ZZ, 0DTL7ZZ, 0DTM7ZZ, 0DTN7ZZ, 8E0W0CZ, 8E0W3CZ, 8E0W4CZ, 8E0W7CZ, 8E0W8CZ
Chemotherapy	CPT: 36640, 51720, 96401, 96402, 96405, 96406, 96409, 96413, 96415, 96416, 96420, 96422, 96423, 96425, 96440, 96446, 96450, 96521, 96522, 96542, 96549<br>ICD-10: Z51.11, Z51.12, 3E0.3305, 3E0.4305, XW0.3351, XW0.33B3, XW0.33C3, XW0.4351, XW0.43B3, XW0.43C3<br>HCPCS: Q0083, Q0084, Q0085, J9000-J9999, J8520, J8521, WW089, WW090, WW091, WW093, WW094, WW096, C9474, Q5107, Q5118, C9025, C9027, C9453<br>Revenue Center: 0331, 0332, 0335
Radiation therapy	CPT: 55860, 55862, 55865, 55875, 77261, 77262, 77263, 77280, 77285, 77290, 77295, 77299, 77300, 77301, 77306, 77307, 77316, 77317, 77318, 77321, 77331, 77332, 77333, 77334, 77336, 77338, 77370, 77372, 77373, 77385, 77386, 77399, 77401, 77402, 77407, 77412, 77417, 77424, 77425, 77431, 77432, 77470, 77499, 77520, 77522, 77523, 77525, 77750, 77761, 77762, 77763, 77770, 77771, 77772, 77778, 77789, 77790, 77799, 79200, 79300, 79440, 79999
**Pancreas cancer codes**	
Diagnosis	ICD-10: C25.0, C25.1, C25.2, C25.3, C25.7, C25.8, C25.9
Biopsy	CPT: 10021, 10022, 38747, 38780, 43231, 43232, 43235, 43238, 43239, 43241, 43242, 43245, 43250, 43251, 43256, 43258, 43259, 43260, 43261, 43268, 43269, 47500, 47505, 47510, 48100, 48102, 48120, 48999, 49000, 49180, 49320, 49321, 49329, 74150, 74160, 74170, 74181, 74182, 74183, 74185, 74320, 74363, 75980, 76360, 76700, 76705, 76942, 76975, 77012, 78811, 78812, 78813, 78814, 78815, 78816, 88172, 88173, 88104, 88305, 88307, 88309<br>HCPCS: G0235, S8085
Surgery	CPT: 48140, 48145, 48146, 48148, 48150, 48152, 48153, 48154, 48155

© 2025 Moen EL et al. *JAMA Network Open*.

	ICD-10: 0FTG0ZZ, 0FTG4ZZ, 0FBG0ZZ, 0FBG3ZZ, 0FBG4ZZ, 0FBG8ZZ
Chemotherapy	CPT: 36640, 51720, 96401, 96402, 96405, 96406, 96409, 96413, 96415, 96416, 96420, 96422, 96423, 96425, 96440, 96446, 96450, 96521, 96522, 96542, 96549<br>ICD-10: Z51.11, Z51.12, 3E0.3305, 3E0.4305, XW0.3351, XW0.33B3, XW0.33C3, XW0.4351, XW0.43B3, XW0.43C3<br>HCPCS: Q0083, Q0084, Q0085, J9000-J9999, J8520, J8521, WW089, WW090, WW091, WW093, WW094, WW096, C9474, Q5107, Q5118, C9025, C9027, C9453<br>Revenue Center: 0331, 0332, 0335
Radiation therapy	CPT: 55860, 55862, 55865, 55875, 77261, 77262, 77263, 77280, 77285, 77290, 77295, 77299, 77300, 77301, 77306, 77307, 77316, 77317, 77318, 77321, 77331, 77332, 77333, 77334, 77336, 77338, 77370, 77372, 77373, 77385, 77386, 77399, 77401, 77402, 77407, 77412, 77417, 77424, 77425, 77431, 77432, 77470, 77499, 77520, 77522, 77523, 77525, 77750, 77761, 77762, 77763, 77770, 77771, 77772, 77778, 77789, 77790, 77799, 79200, 79300, 79440, 79999<br>HCPCS: G6003, G6004, G6005, G6006, G6007, G6008, G6009, G6010, G6011, G6012, G6013, G6014, G6015, G6016

© 2025 Moen EL et al. *JAMA Network Open*.

eFigure 1. Sankey Diagram Visualizing Flow of Radiation, Chemotherapy, and Surgical Procedures to Adjacent and Nonadjacent States

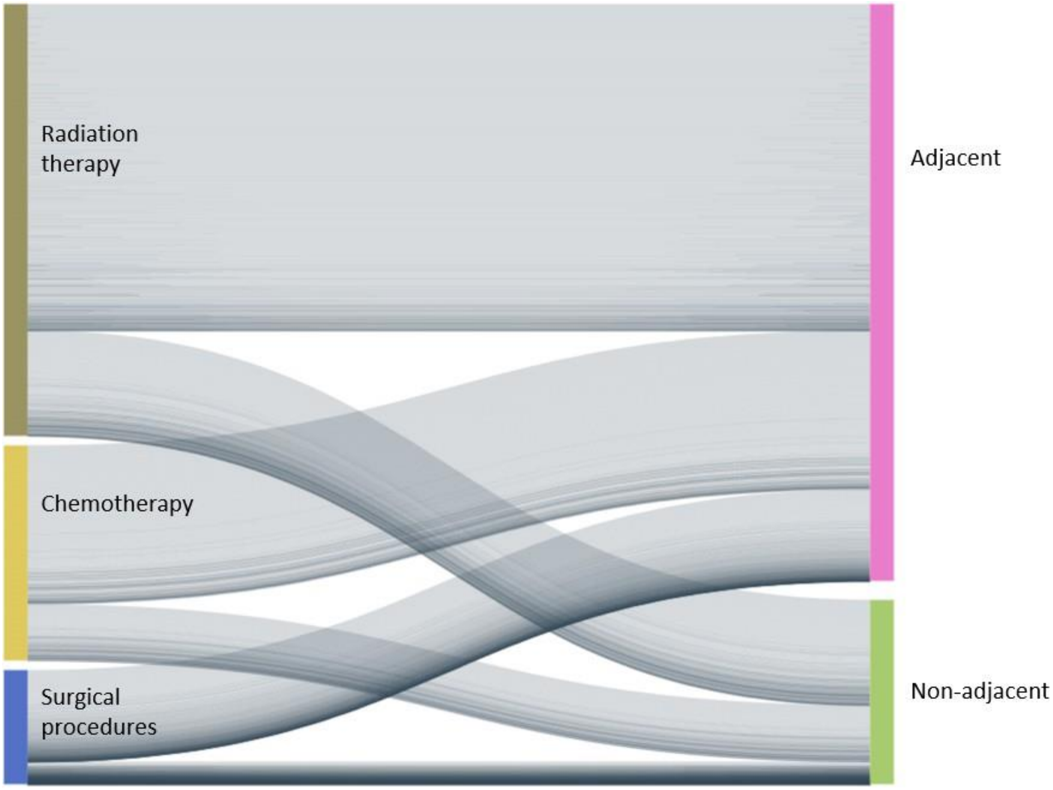

© 2025 Moen EL et al. *JAMA Network Open*.

eFigure 2. Between-State Flows of Cancer Services for All Cancer Services (A), Surgical Procedures (B), Radiation Therapy (C), and Chemotherapy (D)

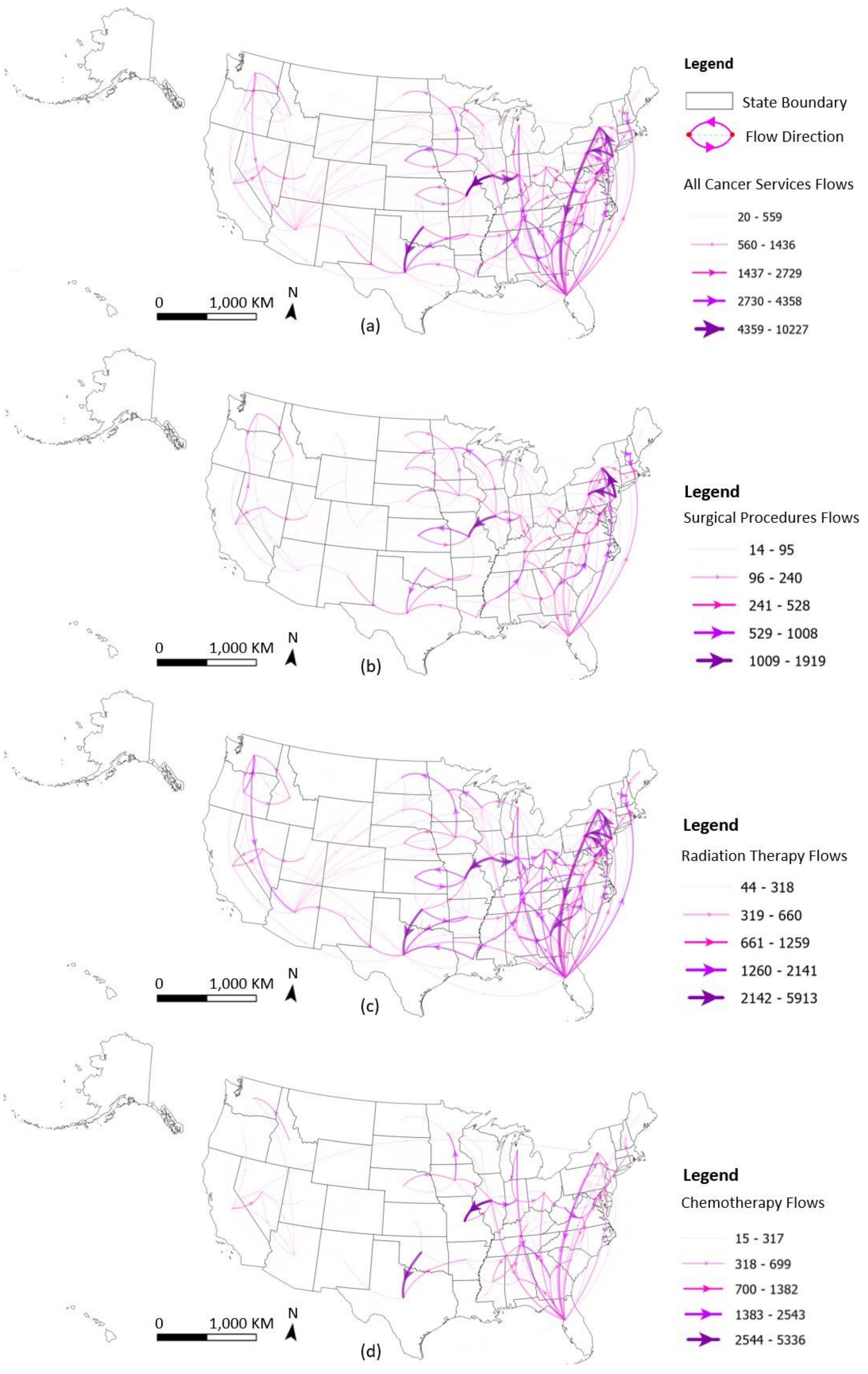

© 2025 Moen EL et al. *JAMA Network Open*.
