# Supplementary material for: Cross-State Travel for Cancer Care and Implications for Telehealth Reciprocity
Source: JAMA Netw Open. 2025 Feb 21;8(2):e2461021. doi: 10.1001/jamanetworkopen.2024.61021 (PMC11846006; doi:10.1001/jamanetworkopen.2024.61021)
Supplement: Supplement 2. — Data Sharing Statement [file jamanetwopen-e2461021-s002.pdf]

Data Sharing Statement

Moen. Cross-State Travel for Cancer Care and Implications for Telehealth Reciprocity. *JAMA Netw Open*. Published February 21, 2025. doi:10.1001/jamanetworkopen.2024.61021

Data
Data available: No

Additional Information
Explanation for why data not available: We used Medicare enrollment and claims data from CMS. We are not allowed to distribute these data, but individuals can access the same base datasets through ResDAC.
